# PyIR: a scalable wrapper for processing billions of immunoglobulin and T cell receptor sequences using IgBLAST

**DOI:** 10.1186/s12859-020-03649-5

**Published:** 2020-07-16

**Authors:** Cinque Soto, Jessica A. Finn, Jordan R. Willis, Samuel B. Day, Robert S. Sinkovits, Taylor Jones, Samuel Schmitz, Jens Meiler, Andre Branchizio, James E. Crowe

**Affiliations:** 1 Vanderbilt Vaccine Center, Vanderbilt University Medical Center, Nashville, TN 37232 USA; 2 Department of Pediatrics, Vanderbilt University Medical Center, Nashville, TN 37232 USA; 3 Department of Pathology, Microbiology, and Immunology, Vanderbilt University, Nashville, TN 37232 USA; 4 San Diego Supercomputer Center, University of California, San Diego, La Jolla, CA 92093 USA; 5 Department of Chemistry, Vanderbilt University, Nashville, TN 37212 USA

**Keywords:** Immune repertoires, Antibody, Illumina, CDR3, IgBLAST

## Abstract

**Background:**

Recent advances in DNA sequencing technologies have enabled significant leaps in capacity to generate large volumes of DNA sequence data, which has spurred a rapid growth in the use of bioinformatics as a means of interrogating antibody variable gene repertoires. Common tools used for annotation of antibody sequences are often limited in functionality, modularity and usability.

**Results:**

We have developed PyIR, a Python wrapper and library for IgBLAST, which offers a minimal setup CLI and API, FASTQ support, file chunking for large sequence files, JSON and Python dictionary output, and built-in sequence filtering.

**Conclusions:**

PyIR offers improved processing speed over multithreaded IgBLAST (version 1.14) when spawning more than 16 processes on a single computer system. Its customizable filtering and data encapsulation allow it to be adapted to a wide range of computing environments. The API allows for IgBLAST to be used in customized bioinformatics workflows.

## Background

The diverse population of rearranged immunoglobulin and T cell receptor (TCR) variable gene sequences within an individual is referred to as their adaptive immune repertoire and is responsible for recognition and neutralization of a potentially unlimited number of pathogenic targets. Next-generation sequencing (NGS) technology has become the ideal method for probing diversity in adaptive immune repertoires [1–5]. With continued advances in NGS technology yielding more sequence reads in less time and at lower cost, the number of researchers interested in analyzing adaptive immune repertoires using different bioinformatics processing pipelines continues to grow [6]. Thus, the demand for efficient and easy-to-use bioinformatics tools for processing and analyzing NGS data from immune repertoire sequencing has never been higher.

Two popular programs for processing immune repertoire sequencing data are IgBLAST [7] and IMGT/HighV-QUEST [8]. IgBLAST is used by several bioinformatic pipelines [9–12]. IMGT/HighV-QUEST is a web utility and is part of the international ImMunoGeneTics (IMGT) information system [13], which provides several web-based tools for immunogenetic analysis. Both tools use template-based nucleotide alignments between curated sets of germline genes and sequencing reads to infer the most plausible recombined germline gene sequences encoding the antibody and to delineate the complementarity-determining regions (CDRs) of the immunoglobulin or T cell receptor (TCR) sequence.

IgBLAST is derived from the well-known BLAST [14] family of sequence alignment and search tools and is available as both a web-based service and a downloadable executable. While the web-based service for IgBLAST is limited by the number of sequences that can be processed at one time, the executable could in principle be used to process millions of sequences. However, the exact number of sequences that can processed is limited by the capacity of the computer hardware used for the task. This limitation is usually not an issue for high-performance computing clusters, but for more conventional workstations, this factor can introduce a barrier to processing data sets with millions of sequences efficiently.

IMGT/HighV-QUEST provides a web-based interface only and accepts a maximum of up to 500,000 sequences per submission. The submission size, although large, can limit the study of very large adaptive immune repertoire sequencing datasets, which are increasingly common. For those cases in which immune repertoire sequences are processed using this tool, results are made available for download in a tab-separated value (TSV) format. IMGT/HighV-QUEST distinguishes itself from all the other adaptive immune repertoire sequencing tools by providing a wealth of information about each processed sequence. However, when processing very large datasets such as sequence sets produced in a single run on current generation NovaSEQ instruments (Illumina), analysis using the IMGT/HighV-QUEST web-portal becomes impractical.

To address the need for processing very large data sets containing immunoglobulin or TCR sequences, we developed a software tool that we call PyIR. The software is a minimally dependent Python3 wrapper and library for IgBLAST [7] and can scale to process up to 1 billion sequences. Its basic functionality splits the input FASTX (FASTQ or FASTA file) file and performs batch execution on chunks of sequences. This approach avoids having to read all data in memory at once, allowing efficient processing of very large data sets on modest size workstations with multiple CPUs. PyIR parses all of the IgBLAST-generated fields from the web-based file format into fields that comply with Adaptive Immune Receptor Repertoire (AIRR) recommendations [15] and then outputs the results into a JSON file format. PyIR also provides options for sequence quality filtering that can be invoked from the command line. These sequence filters allow the user to remove poor quality data after IgBLAST processing. We also have included an application programming interface (API) with PyIR that allows users to incorporate this tool directly into their own Python scripts. PyIR will be a useful computational tool for the AIRR community [15] by allowing those researchers with minimal Python programming experience to easily interface with IgBLAST.

## Implementation

PyIR is available for Linux or OSX (Darwin) environments running Python 3.6 or higher. Installation is managed by the Python pip3 module, which also installs PyIR dependencies as needed. PyIR ships with IgBLAST (version 1.14) and users implement the software with their own executable and germline gene databases. We provide current germline genes from IMGT in this distribution, but we recommend that users download the most up-to-date version of the germline genes from IMGT at: http://www.imgt.org.

PyIR will run on machines with modest processing power and memory. For example, we have used a MacBook Pro with a 2.7GHz 2 core/4 thread i5 CPU and 8GB RAM (Apple) for this type of analysis. However, some consideration must be made for disk space, since PyIR stores runtime data as temporary files. Output files generated when running PyIR using standard options are expected to be 4 to 5 times larger than the input FASTX files.

## Results and discussion

### PyIR processing time

The critical enhancement in PyIR versus multithreaded IgBLAST (version 1.14) is the addition of file chunking. At the beginning of execution, PyIR estimates the number of input sequences based on the FASTX file size and then splits the file into ‘chunks’ that are stored as temporary files on disk. The default PyIR behavior is to balance the number of sequences per chunk with the number of CPU threads available and the size of the input file, but the user also can manually override this option to set a specified chunk size. PyIR then distributes chunks continuously to available processors for IgBLAST to process.

**Fig. 1:**
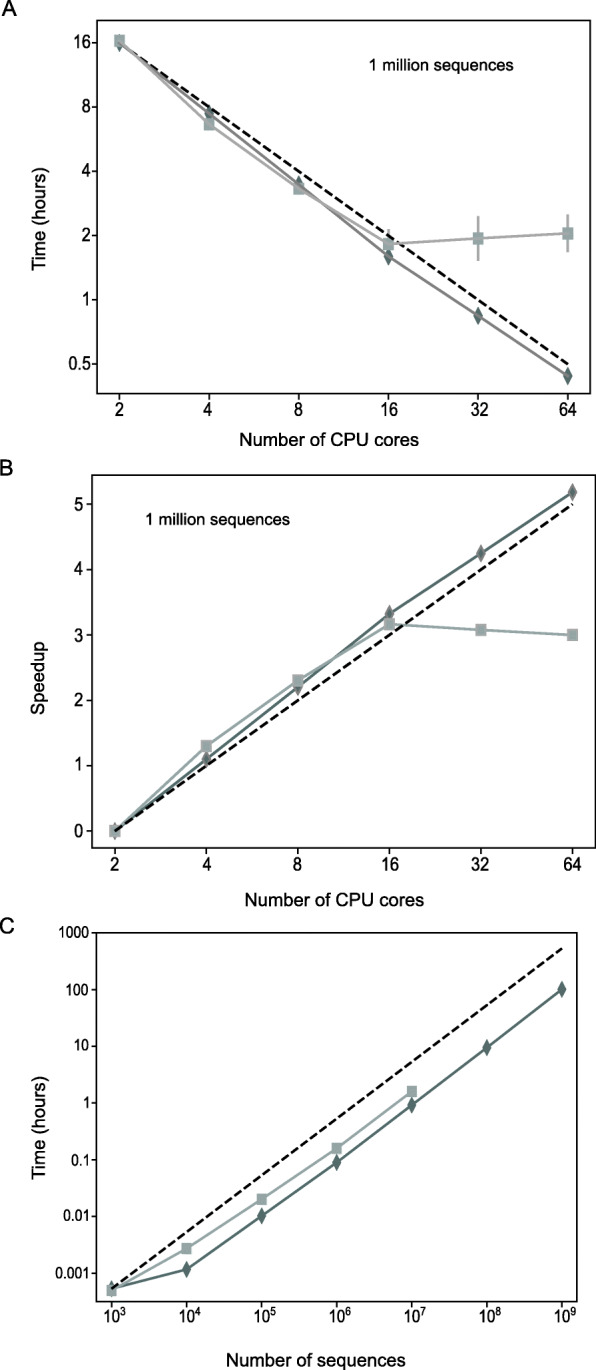
Multiprocessing performance for PyIR and multithreaded IgBLAST (version 1.14). **a** One million synthetic immunoglobulin sequences were used to time PyIR (dark grey, ♦) against multithreaded IgBLAST (version 1.14) (grey, ■) as a function of the number of processes. Idealized timings are shown as a black dashed line. Average timings were measured over the three trial runs for 1 million sequences and computed separately for both IgBLAST and PyIR. Standard deviations appear as error bars for both methods. X and Y axes are in log_2_ space. **b** The speedup of PyIR relative to multithreaded IgBLAST (version 1.14) as a function of the number of simultaneous processes. Timings were done on a workstation equipped with 4 Opteron 6278 hyper-threaded 8-core processors for a total of 64 CPU threads using the average timings from (**a**). The X and Y axes are in log_2_ space. **c** One billion synthetic immunoglobulin sequences were used to determine the speedup PyIR achieved over multithreaded IgBLAST (version 1.14) as a function of the number of sequences. Idealized speedups are shown as a black dashed line. Timings were done on a workstation equipped 4 Xeon Platinum 8280 hyperthreaded 28-core processors for a total of 224 CPU threads. X and Y axes are in log_10_ space

To test the processing efficiency of PyIR, we processed 1 million synthetic immunoglobulin sequences [1] using PyIR and multithreaded IgBLAST (version 1.14) on a workstation with 4 hyperthreaded 8-core Opteron 6278 processors. Both PyIR and multithreaded IgBLAST (version 1.14) showed linear scaling out to 16 processes (Fig. 1a). However, when more than 16 processes are spawned only PyIR maintains this linear scaling out to 64 processes (Fig. 1a). We also looked at speedup, defined here as the performance gained by spawning additional processes. We computed speedup by normalizing the time in hours to process 1 million (or 1 billion) sequences by the time it takes to process 1 million (or 1 billion) sequences using two processes. Both PyIR and multithreaded IgBLAST (version 1.14) show linear speedups out to 16 processes, but when more than 16 processes were spawned, IgBLAST did not show speedup (Fig. 1b). Thus, PyIR maintains linear scaling beyond 16 processes using all of the threads available from the hardware.

We also tested the ability of PyIR to process very large data sets (i.e., cDNA sequence data sets from analysis on a NovaSEQ instrument) using file chunking. We ran a separate set of trials on a workstation with 4 hyperthreaded 28-core Xeon Platinum 8280 processors, clocking the total time required to analyze a series of FASTA files containing from 1 thousand to 1 billion synthetic immunoglobulin sequences. Multithreaded IgBLAST (version 1.14) failed at around 70 million sequences, while PyIR ran to completion (Fig. 1c). These results demonstrate the efficiency of PyIR and the underlying file chunking method to process very large data sets.

PyIR also contains options for sequence filtering, allowing users to remove sequences with stop codons or those that are out-of-frame according to IgBLAST. For example, we typically filter out sequences that: 1) fail to meet our minimum V and J gene assignment expectation value (E-value) threshold of 1.0E-6, 2) contain a stop codon in the translated nucleotide sequence, 3) are out-of-frame, or 4) lack a complementarity-determining region 3 (CDR3) region. These sequence filters can be enabled or disabled individually and any non-boolean filters (i.e., E-value filter) can be set directly by the user. We also have included a Phred score filter that can be applied to the CDR3 region of a sequence. This filter removes any sequence with a nucleotide in the CDR3 region with a Phred score below some user-defined threshold. The filtering options can be extended by users directly within PyIR or through the application programming interface (API).

### PyIR application programming interface (API)

The API interface of PyIR can be used to call PyIR functions from within a Python script. This feature allows users to extend the functionality of PyIR or to use PyIR within a user’s own adaptive immune repertoire processing pipeline. Several published methods for processing immune repertoire data lack an accessible API, which is limiting for some experimental immunologists who may be new to scripting in Python. An example of how one would incorporate PyIR within a Python script is given directly below:

**from pyir import PyIR**

**FILE = 'test.fasta'**

**pyir = PyIR (query=FILE)**

**result = pyir.run()**

**print (result)**

In this example, PyIR is imported directly into Python and used to process a FASTA formatted file called *test.fasta*. The Python object in the variable *result* is a Python dictionary containing all the parsed fields from the web-based output of IgBLAST. We provide five examples showing how one could make use of PyIR directly within a Python script (see http://github.com/crowelab/PyIR). The fifth example shows how one could use the PyIR API to generate a histogram of CDR3 lengths (see Additional file 1).

### PyIR output

After processing and sequence filtering, PyIR can return a zipped JSON file, a tab separated value file (TSV) or a Python dictionary (if PyIR is used as an API). We do note that the JSON output file can be large since PyIR stores the parsed results from the three best alignments. The user has the option of storing only the single best alignment, which reduces the size of the JSON file. Our primary focus for using JSON as the preferred output format was to allow for easy insertion into a MongoDB database. Several recent studies have been published that contain large adaptive immune repertoire sequencing datasets [1, 2, 16]. Facilitating the ability to process and store these data sets locally into an industry standard database such as MongoDB or MariaDB motivated use of the JSON format.

## Conclusion

PyIR was designed to extend the functionality of IgBLAST to allow for processing of very large datasets (> 100 million antibody or TCR recombined variable gene sequences). In terms of processing efficiency, we found that PyIR scaled linearly with the number of processes out to 1 billion sequences. Multithreaded IgBLAST (version 1.14) also scaled linearly but failed at around 70 million sequences. Our benchmarks suggest that PyIR can process about 2 million sequences per hour on a modest 64-core server and can process roughly 10 million sequences per hour on a 112-core machine that sits on the premium end of enterprise hardware. The API provides novice Python programmers with the ability to interface directly with IgBLAST and to explore using this tool in their own bioinformatics workflows. We do note that PyIR is not the only method for processing immune repertoire sequencing [17]. However, PyIR is easily adaptable and uses IgBLAST which has been extensively benchmarked against other methods [18]. We expect that PyIR will find use among the Adaptive Immune Receptor Repertoire (AIRR) Community.

### Availability and requirements

Project name: PyIR

**Project homepage:**http://github.com/crowelab/PyIR

**Archived version:**10.5281/zenodo.3862746

**Operating systems:** Linux, UNIX, OSX (Darwin)

**Programming languages**: Python

**Other requirements**: None

**License:** Free to academics

**Any restrictions to use by non-academics:** Yes; non academics should contact the author for permission to use the software or license options for incorporation into software that is being sold for profit.

## Supplementary information

**Additional file 1.** Generating CDR3 length distributions with the PyIR API. Synthetic sequence data from Briney et al., was used to demonstrate the use of PyIRs API in generating a CDR3 length distribution.

## Data Availability

The datasets and source code supporting the conclusions of this article are available in the GitHub repository, http://github.com/crowelab/PyIR.

## Abbreviations

NGS: Next-generation sequencing
CDR3: Complementarity-determining region 3
JSON: JavaScript Object Notation
API: Application Programming Interface
CLI: Command-line interface
FASTA: File format for representing nucleotide sequence
FASTQ: File format for storing nucleotide sequence and quality scores
