# Supplementary material for: PyIR: a scalable wrapper for processing billions of immunoglobulin and T cell receptor sequences using IgBLAST
Source: BMC Bioinformatics. 2020 Jul 16;21:314. doi: 10.1186/s12859-020-03649-5 (PMC7364545; doi:10.1186/s12859-020-03649-5)
Supplement: Supplementary file 1 — Additional file 1. Generating CDR3 length distributions with the PyIR API. Synthetic sequence data from Briney et al., was used to demonstrate the use of PyIRs API in generating a CDR3 length distribution. [file 12859_2020_3649_MOESM1_ESM.pdf]

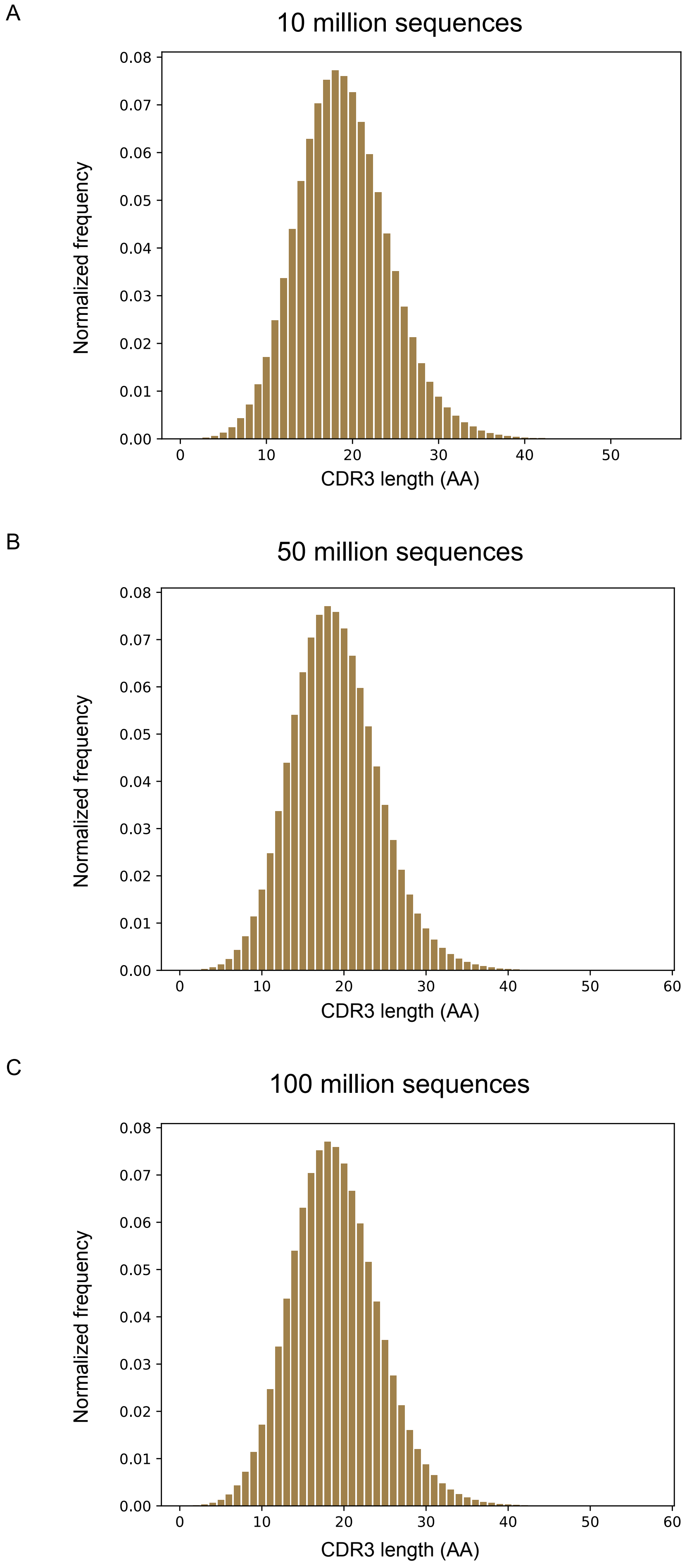

Supplementary Figure 1: **Generating CDR3 length distributions with PyIR's API**. All data used in generating histograms come from synthetic heavy chain sequence data used in the Briney *et al.*, study. All CDR3 length distributions were nornalized by the total number of productive sequences after processing and filtering sequences with PyIR. (A) 10 million sequences (B) 50 million sequences and (C) 100 million sequences.
